# *Myo*-Inositol Supplementation Alleviates Cisplatin-Induced Acute Kidney Injury via Inhibition of Ferroptosis

**DOI:** 10.3390/cells12010016

**Published:** 2022-12-21

**Authors:** Huiyue Qi, Fei Deng, Yinghuai Wang, Hao Zhang, Yashpal S. Kanwar, Yingbo Dai

**Affiliations:** 1 Department of Urology, Guangdong Provincial Key Laboratory of Biomedical Imaging, The Fifth Affiliated Hospital of Sun Yat-sen University, Zhuhai 519000, China; 2 Department of Urology, The Second Xiangya Hospital at Central South University, Changsha 410000, China; 3 Department of Pathology & Medicine, Northwestern University, Chicago, IL 60611, USA

**Keywords:** *myo*-inositol, cisplatin, acute kidney injury, ferroptosis

## Abstract

*Myo*-inositol, a carbocyclic sugar, is believed to be relevant to renal pathobiology since the kidney is the major site for its catabolism. Its role in acute kidney injury (AKI) has not been fully investigated. Ferroptosis, a unique form of regulated cell death, is involved in various types of renal injuries. The relevance of *myo*-inositol with respect to the process of ferroptosis has not been explored either. Herein, our current exploratory studies revealed that supplementation of *myo*-inositol attenuates cisplatin-induced injury in cultured Boston University mouse proximal tubular (BUMPT) cells and renal tubules in vivo. Moreover, our studies unraveled that metabolic parameters pertaining to ferroptosis were disrupted in cisplatin-treated proximal tubular cells, which were seemingly remedied by the administration of *myo*-inositol. Mechanistically, we noted that cisplatin treatment led to the up-regulation of NOX4, a key enzyme relevant to ferroptosis, which was normalized by the administration of *myo*-inositol. Furthermore, we observed that changes in the NOX4 expression induced by cisplatin or *myo*-inositol were modulated by carboxy-terminus of Hsc70-interacting protein (CHIP), an E3 ubiquitin ligase. Taken together, our investigation suggests that *myo*-inositol promotes CHIP-mediated ubiquitination of NOX4 to decelerate the process of ferroptosis, leading to the amelioration of cisplatin-induced AKI.

## 1. Introduction

Acute kidney injury (AKI) is an ominous clinical–pathologic state associated with the abrupt loss of renal functions and consequential complications. It is estimated that more than 20–50% of hospitalized patients are affected by various episodes of AKI [1]. Ironically, even one incident of AKI confers patients to a high risk of chronic kidney injury during their convalescence [2]. The etiology of AKI is complex, and it may be due to sepsis [3], ischemia/reperfusion injury [4], immunologic disorders [5], or the administration of nephrotoxic agents [6]. Cisplatin is a potent first-line of chemotherapeutic drug for treating various neoplasms, but its administration in hospital settings is hampered due to its untoward nephrotoxicity. Previous investigations indicated that about 30% of cisplatin-treated patients (at the dose of 50–100 mg/m^2^) developed AKI [7]. Even those who received rehydration therapy before the administration of cisplatin still presented with variable degrees of renal injury. Due to its dire nephrotoxicity, cisplatin-induced AKI is exclusively used as a prototype for research investigations [8]. Once inside the bloodstream, cisplatin filters freely across the glomerular capillaries, and then gets reabsorbed into the proximal tubular cells, leading to acute tubular necrosis (ATN) [9]. Various mechanisms, such as inflammation [10], autophagy [11], apoptosis [12], and redox injury [13], have been proposed in the pathogenesis of cisplatin-induced AKI, but exact pathogenetic processes leading to this form of injury still remain somewhat elusive.

Our recent studies highlighted the initial evidence that ferroptosis is an integral process in the pathogenesis of cisplatin-induced AKI [14], and it was very soon validated by two other groups of investigators [15,16]. Ferroptosis is a unique form of non-apoptotic regulated cell death that is characterized by excessive intracellular free iron and overwhelming generation of iron-catalyzed lipid peroxides [17]. The pathogenesis of ferroptosis is rather complex, and it includes metabolic disruptions encompassing lipid, iron, and amino acids [18]. Ferroptosis can be seen in various pathological processes involving diverse organ systems, such as ischemia/reperfusion-induced brain injury, doxorubicin-induced cardiomyopathy, alcohol-related hepatic disorders, and chemotherapeutically-induced cancer cell death [17]. In the realm of the kidney, ferroptosis has been observed in the progression of ischemia/reperfusion-, folic acid-, oxalate-, rhabdomyolysis-, and cisplatin-induced tubular injuries [19,20,21]. In spite of the recent intensive investigations using various model systems to delineate the mechanisms involved in ferroptosis, the precise cellular events leading to renal ferroptosis essentially remain yet to be clearly defined.

*Myo*-inositol, a carbocyclic sugar, is ubiquitously present in different organs, and it is involved in various bio-processes as free form or its conjugated phosphate derivatives [22]. Interestingly, *myo*-inositol metabolism is closely associated with the biophysiology of the kidneys. In mammals, *myo*-inositol is principally synthesized by the kidneys. An enzyme known as *myo*-inositol oxygenase (MIOX), which is present exclusively in the kidney, catabolizes *myo*-inositol in the renal proximal tubular cells [23]. Incidentally, *myo*-inositol at times is given as a dietary supplement, for instance, to patients with polycystic ovarian syndrome (PCOS) [22]. Our previous studies suggest that MIOX overexpression exacerbates cisplatin-induced AKI by accelerating ferroptosis [14]. Moreover, MIOX gene deletion leads to increased levels of *myo*-inositol in the bloodstream and kidneys of mice [24]. However, the role of *myo*-inositol in cisplatin-induced AKI has yet to be defined. The aim of this investigation was to assess the therapeutic potential of *myo*-inositol in terms of its benefits in the amelioration of cisplatin-induced AKI and its relevance to ferroptosis.

## 2. Materials and Methods

### 2.1. Cell Culture Studies

BUMPT (Boston University mouse proximal tubular) cells, the mouse proximal tubular cell line, were purchased from ATCC. Cells were maintained in a culture medium containing DMEM, 10% FBS, and antibiotics in a humidified chamber with 5% CO_2_. For cell treatment, cells were seeded into 6-well plates or 96-well plates containing cell culture medium with 2% FBS. Cells were treated with 20 μM cisplatin (SigmaAldrich, catalog # P4394) for 6–20 h for in vitro challenge. For *myo*-inositol treatment, 10 mM *myo*-inositol (Sigma Aldrich, catalog # I5125) was used to prime the cells 4 h before the administration of cisplatin. Then, 0.2 μM RSL3 (Selleck, catalog # S8155) was used to treat BUMPT cells for 12 h to induce ferroptosis. For the manipulation of CHIP expression, CHIP overexpressing lentivirus (CHIP-OV) and CHIP knockdown lentivirus (CHIP-KD) were used. The lentivirus-modified BUMPT cells were screened following treatment with puromycin to obtain various stable transfectants.

### 2.2. Animal Studies 

Eight-to-ten-weeks-old C57BL/6J mice weighing 20–24 mg were purchased from Guangdong Medical Laboratory Animal Center. The rules of the Guide for the Care and Use of Laboratory Animals published by the US National Institutes of Health were strictly followed. Additionally, protocols enforced by the Institutional Animal Care and Use Committees of Sun Yat-sen University were used. Mice were divided into a control group, cisplatin-treated group, and cisplatin + *myo*-inositol-treated group, which were treated with cisplatin or *myo*-inositol accordingly. Mice received intraperitoneal injection of cisplatin at a dose of 25 mg/kg, and they were sacrificed by cervical dislocation three days later. The kidneys and serum samples of the mice were harvested for various studies. For *myo*-inositol supplementation, mice were intraperitoneally injected with *myo*-inositol (360 mg/kg/d, as previously reported [25]) for 7 days before cisplatin administration till the day of sacrifice. 

### 2.3. Cell Morphology Analysis

For light microscopic studies, treated cells were examined by phase-contrast microscopy. For H&E staining of the cells, cells were fixed with paraformaldehyde for 15 min, and they were then subsequently stained with hematoxylin (3 min) and eosin (45 s). The stained cells were evaluated by light microscopy.

### 2.4. Cell Survival Measurement

MTT assay was used for the examination of cellular survival rate. Briefly, about 5 × 10^3^ cells were seeded into 96 well plates, and they were then treated as indicated above after overnight incubation. The culture medium was replaced with fresh medium containing 1 mg/mL Thiazolyl Blue Tetrazolium Bromide (Sigma-Aldrich, catalog # M5655), and cells were maintained for another 4–6 h. The medium was then carefully removed and 150 μL DMSO was added. The plate was shaken for 5 min before the examination by microplate reader at a wavelength of 495 nm.

### 2.5. Retrieval of RPKM Values

For the initial evaluation of gene expression, the RPKM value was obtained from the NCBI website: https://www.ncbi.nlm.nih.gov/mesh (accessed on 22 October 2022). “Gene” was selected in the dialog box on the left side, and gene name was entered. After searching, gene name in different species was revealed, and the expression of the gene in a certain species can be obtained in the link.

### 2.6. Renal Morphological Analysis

H&E staining was used for renal morphology studies. Paraffin sections of 4 μm thickness were de-paraffinized and rehydrated following routine protocols. The sections were then stained with hematoxylin for 3 min and eosin for another 30 s. The sections were dehydrated and coverslip-mounted for microscopic evaluation. PAS staining was also employed for morphologic evaluation using Periodic Acid Schiff Staining Kit (Abcam, catalog # ab150680). The sections were incubated with periodic acid solution for 10 min after de-paraffinization and rehydration. Subsequently, they were immersed in Schiff solution for 30 min after rinsing with water. The sections were then washed with water and stained with hematoxylin (3 min). They were then rinsed with water and incubated with bluing reagent (30 s). Finally, the sections were dehydrated and mounted for microscopic examination.

### 2.7. C11 Staining

For the evaluation of lipid ROS generation in vitro, C11 staining by C11 BODIPY 581/591 kit (ThermoFisher, catalog # C10445) was used in our study. After the treatment, cell culture media was removed, and 10 μM Image-iT^®^ Lipid Peroxidation Sensor (Component A) was applied to the cells for 30 min at 37 °C. PBS solution was then used to wash the cells three times, and the cells were immediately examined by fluorescence microscopy.

### 2.8. FerroOrange Staining

For the detection of labile iron in vitro, FerroOrange (DOJINDO, catalog # F374) was used to stain BUMPT cells. Briefly, FerroOrange was initially dissolved in DMSO for stocking (at the concentration of 1 mM). A total of 2 μM of working solution (diluted in fetal bovine serum-free medium) was used to stain the cells for 30 min. The cells were finally evaluated by fluorescence microscopy.

### 2.9. Renal Function Evaluation

Serum creatinine was measured using creatinine assay kit (BioAssay Systems, catalog # DICT-500). Briefly, blood samples were collected from retro-ocular vein plexus after anesthesia, which were then maintained at room temperature for 60 min to assure coagulation. After that, coagulated blood samples were centrifuged at 3500 rpm for 15 min until the serum was clear in the upper layer, which was collected for further investigations. The serum samples were mixed with the reaction reagent, and the mixtures were read by a microplate reader (510 nm) at 0 min and 5 min. The difference between two readings indicated the concentration of serum creatinine. Serum BUN levels were also evaluated by using BUN kit (Applygen Technologies, catalog E2020). The serum samples were added to a reaction reagent, and the mixtures were read at 540 nm. The standards were used for calculation and data analyses.

### 2.10. qRT-PCR Studies

About 20 mg of renal cortex was homogenized in TRIzol reagent (Invitrogen, catalog 15-596-026), and the mRNA was extracted by following routine protocols. RNA of 1 μg was used to reverse-transcribe into cDNA with the PrimeScript^TM^ RT reagent kit (Takara). For quantitative measurement, ChamQ^TM^ Universal SYBR^®^ qPCR Master Mix (Vazyme) was used, and the reaction was carried out in an ABI PRISM 7900 Sequence Detector System (Applied Biosystems). The calculated values were compared with GAPDH or β-actin for statistical analysis. The primers (from 5′ to 3′) used in our study were: NOX4-F: GAAGGGGTTAAACACCTCTGC, NOX4-R: ATGCTCTGCTTAAACACAATCCT; KIM-1-F: AGTCAGCATCTCTAAGCGTGG, KIM-1-R: ATGTAGATGTTGTCTTCAGCTCG; NGAL-F: GAGCTACAATGTGCAAGTGGC, NGAL-R: GCTCCTTGGTTCTTCCATACAG; ACSL4-F: CTCACCATTATATTGCTGCC TGT; ACSL4-R: TCTCTTTGCCATAGCGTTTTTCT; PTGS2-F: TTCCAATCCATGTCA AAACCGT; PTGS2-R: AGTCCGGGTACAGTCACACTT; β-actin-F: GGCTGTATTCCC CTCCATCG, β-actin-R: CCAGTTGGTAACAATGCCATGT; and GAPDH-F: ACTCTTC CACCTTCGATGCC, GAPDH-R: TGGGATAGGGCCTCTCTTGC.

### 2.11. Western Blotting Procedures

Renal cortex or BUMPT cells were homogenized, and the homogenates were centrifuged for the collection of the supernatants. The protein concentration was determined by BCA assay (Beyotime, catalog P0012S). The samples with equal amounts of protein were then subjected to SDS-PAGE analysis and the fractionated proteins were transferred to PVDF membranes (0.22 μm). The membranes were immersed in 5% milk for 1 h to block the background. They were incubated with diluted primary antibodies overnight at 4 °C. The primary antibody used included: anti-NCOA4 (Bethyl Laboratories, catalog # A302-272A; 1:1000), anti-FTH1 (Cell Signaling Technology, catalog # 3998S; 1:1000), anti-GPX4 (Affinity Biosciences, catalog DF6701; 1:1000), anti–NOX4 (Abcam, catalog # ab133303; 1:2000), anti–CHIP (Abcam, catalog # ab134064; 1:2000), anti-β-actin (proteintech, catalog # 66009-1-Ig; 1:5000) and anti-GAPDH (proteintech, catalog # 60004-1-Ig, 1:1000). The membranes were washed with TBST and incubated with secondary diluted antibodies (1:1000) for 1–2 h. Finally, the membranes were washed and subjected to evaluation by ECL chemiluminescence detector.

### 2.12. Immunofluorescence (IMF) Studies 

Four μm thick paraffin slide sections were de-paraffinized, rehydrated, permeabilized, and washed with PBST using routine protocols. For antigen retrieval, EDTA buffer (solarbio, catalog # C1034-100) was used to immerse the sections, and they were placed in a pressure cooker. The sections were heated in the cookers for 10 min after boiling, which were then slowly cooled down to room temperature. The sections were then blocked with 10% goat serum in TBST for 1 h at room temperature. The sections were incubated with 4-HNE antibody (Abcam, catalog # ab46545; 1:200) at 4 °C overnight. The sections were washed and incubated with secondary antibody conjugated with FITC for 1 h at room temperature. Finally, the sections were DAPI stained, washed, and coverslip-mounted for fluorescence microscopic evaluation.

### 2.13. Immunohistochemical Staining (IHC)

The same protocol as of IMF, i.e., de-paraffinization, rehydration, permeabilization, and antigen retrieval were applied to 4 μm-thick paraffin-embedded sections for IHC. Endogenous peroxidase was quenched by H_2_O_2_ solution (3%). The sections were rinsed, and they were immersed in 10% goat serum in TBST for 1 h for blocking the background. The sections were incubated with primary diluted antibody at 4 °C overnight. The primary antibodies used were anti-NOX4 (Abcam, catalog # ab133303; 1:200) and anti-CHIP (Abcam, catalog # ab134064; 1:200). The sections were then washed and incubated with secondary diluted antibody for 1 h at room temperature. After three washes with PBS, DAB staining was applied, and the sections were rewashed, dehydrated, and cover-slip mounted for light microscopic evaluation.

### 2.14. Measurement of ROS Generation

The ROS generation in BUMPT cells was detected by H2-DCFDA staining. Briefly, the treated cells were stained with 5 μM H2-DCFDA (ThermoFisher, catalog # D399) solution in PBS for 15 min at room temperature. The cells were washed and evaluated by fluorescence microscopy. The mitochondrial ROS generation was evaluated by Mito-sox staining. Cells after various treatments were stained with 5 μM Mito-sox (ThermoFisher, catalog # M36008) for 30 min and subjected to microscopic examination. The ROS generation in renal sections was measured by DHE staining. Briefly, the sections were de-paraffinized and rehydrated as mentioned above. Then, 20 μM dihydroethidium (Sigma-Aldrich, catalog # D7008) was applied to the sections for 15 min at room temperature. The sections were rinsed and evaluated by microscopy.

### 2.15. Statistics

Graphpad prism 8.0 was used for statistical analyses. Data were expressed as mean ± standard deviation. Student’s *t*-test was used for the difference analysis of two groups, and one-way ANOVA with Dunn’s multiple comparisons was used for the difference analysis of multiple samples. *p* value less than 0.05 was regarded as statistically significant.

## 3. Results

### 3.1. Myo-Inositol Treatment Attenuates Cisplatin-Induced Cellular Injury in BUMPT Cells

**Figure 1:**
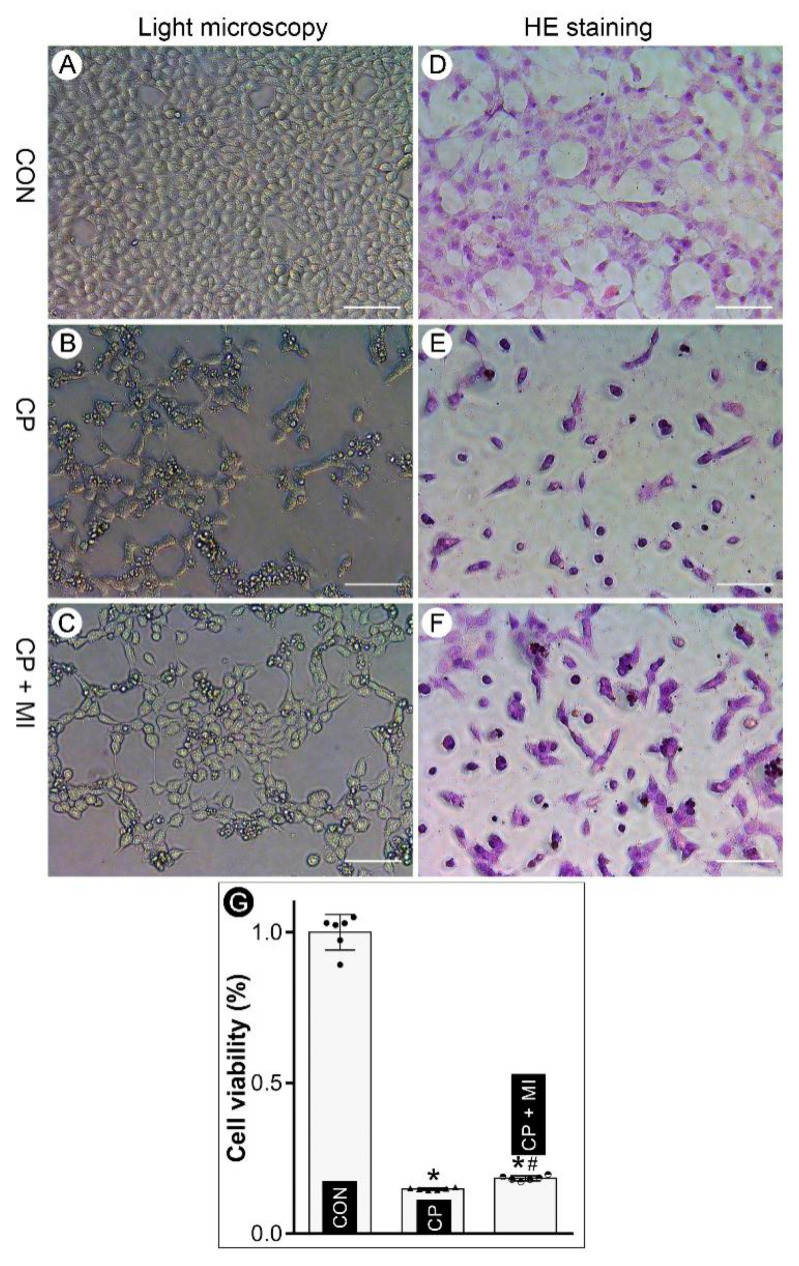
***Myo*-inositol alleviates cisplatin-induced BUMPT cell death.** (Panels (**A**–**C**)) Phase-contrast microscopy revealed that cisplatin (CP) treatment caused a marked deterioration in the morphology and decrease in the number of surviving BUMPT cells, while *myo*-inositol (MI) treatment led to a considerable improvement in morphology and cell number. (Panels (**D**–**F**)) H&E staining revealed that the treatment of *myo*-inositol ameliorates cisplatin-induced cell injury. (Panel (**G**)) MTT assay indicated that *myo*-inositol improved the viability of BUMPT cells which was markedly reduced following cisplatin treatment (n = 6). * *p* < 0.05 compared with control group, ^#^ *p* < 0.05 compared with CP group, scale bar: 100 μm.

BUMPT cell line was used to evaluate the beneficial effects of *myo*-inositol in in vitro studies. Our initial MTT assay showed that 10 mM *myo*-inositol had conceivable protection in cisplatin-induced injuries, which was used for further investigations (Supplemental Figure S1). Cell morphology was evaluated by phase-contrast microscopy and hematoxylin and eosin (H&E) staining. Following cisplatin treatment, the number of live BUMPT cells was decreased, and many deformed cells (characterized by cellular shrinkage and unstained nucleus) were also observed (Figure 1B,E vs. Figure 1A,D). Interestingly, the *myo*-inositol treatment attenuated the aberrant alterations in cisplatin-treated BUMPT cells (Figure 1C,F vs. Figure 1B,E). Cell survival rate was also investigated by MTT assay. The data revealed that cell death was induced in the presence of cisplatin, which was partially mitigated by *myo*-inositol treatment (Figure 1G). Overall, the above-mentioned results suggested that *myo*-inositol treatment partially shields BUMPT cells against cisplatin-induced injurious effects.

### 3.2. Myo-Inositol Supplementation Alleviates Cisplatin-Induced AKI

**Figure 2:**
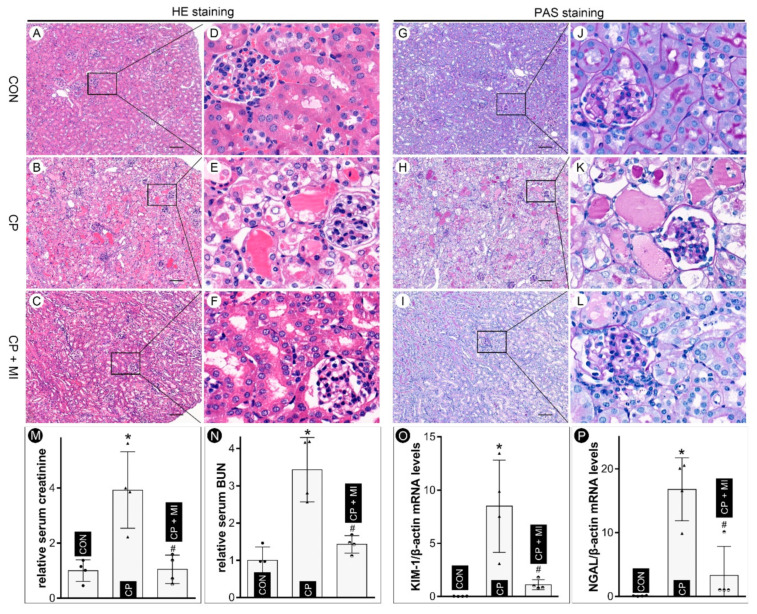
***Myo*-inositol attenuates cisplatin-induced AKI in mice.** (Panels (**A**–**F**)) H&E staining showed that *myo*-inositol treatment alleviated cisplatin-induced renal damage. (Panels (**G**–**L**)) PAS staining revealed that cisplatin caused severe cellular damage to the tubules, which was mitigated by *myo*-inositol supplementation. (Panels (**M**,**N**)) Analyses of serum creatinine showed that *myo*-inositol attenuated cisplatin-induced increase in serum creatinine and BUN (n = 4). (Panels (**O**,**P**)) qRT-PCR analyses revealed that *myo*-inositol normalized the cisplatin-induced increase in the mRNA levels of KIM-1 and NGAL (n = 4). * *p* < 0.05 compared with control group, ^#^ *p* < 0.05 compared with CP group, scale bar: 100 μm.

In order to evaluate the role of *myo*-inositol in cisplatin-induced injury in vivo, daily intraperitoneal injection of *myo*-inositol was administered to C57BL/6J mice. The renal morphologic changes were assessed after H&E staining. Severe proximal renal tubular damage (characterized by tubular dilatation, cast formation, interstitial edema, cellular vacuolization, and focal nuclear drop out) was observed in kidneys of cisplatin-treated mice, as compared with the controls (Figure 2B,E vs. Figure 2A,D). Interestingly, the renal morphological disruption was alleviated by the *myo*-inositol supplementation (Figure 2C,F vs. Figure 2B,E). These morphologic changes were also readily observed in tissue sections stained with periodic acid–Schiff (PAS). In addition, PAS-stained sections revealed that cisplatin treatment led to notable brush border disruption and PAS-positive hyaline casts in proximal tubular lumina (Figure 2H,K vs. Figure 2G,J). These morphologic aberrations were mitigated with the treatment of *myo*-inositol (Figure 2I,L vs. Figure 2H,K). Analysis of renal functions revealed notable deterioration in their parameters. The data indicated that cisplatin treatment led to a remarkable rise in serum creatinine and blood urea nitrogen (BUN) (Figure 2M,N). These physiological changes were largely normalized in mice co-treated with cisplatin and *myo*-inositol. The changes in tubular specific injury markers, i.e., KIM-1 and NGAL, were also investigated. The qRT-PCR analysis indicated a marked increase in KIM-1 and NGAL mRNA levels in cisplatin-treated kidneys, which were normalized with the supplementation of *myo*-inositol (Figure 2O,P). Taken together, these data suggest that the *myo*-inositol treatment ameliorates cisplatin-induced AKI.

### 3.3. Myo-Inositol Inhibits NOX4-Driven Ferroptosis to Mitigate Cisplatin-Induced BUMPT Cell Injuries

**Figure 3:**
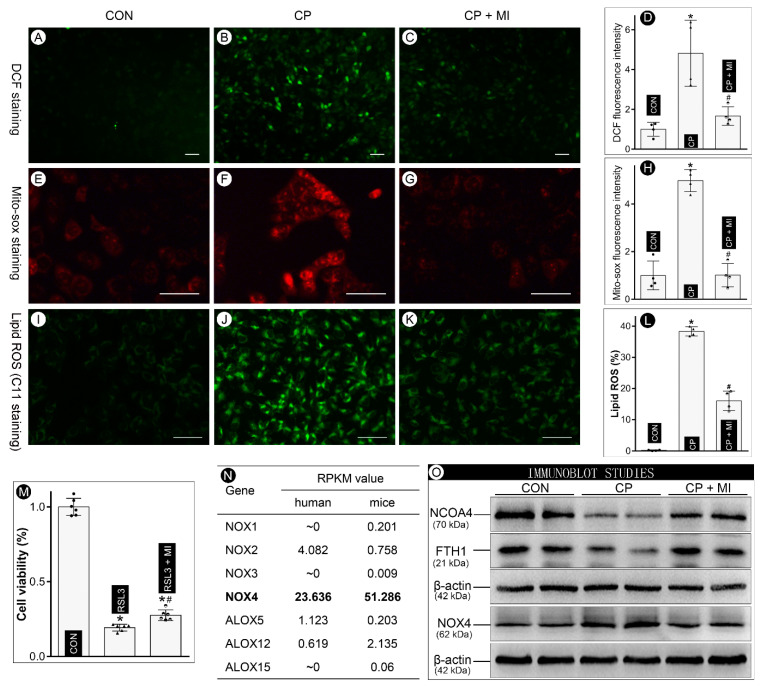
***Myo*-inositol decelerates ferroptosis in cisplatin-treated BUMPT cells.** (Panels (**A**–**D**)) DCF staining showed that *myo*-inositol alleviated cisplatin-induced cytoplasmic ROS generation in BUMPT cells (n = 4). (Panels (**E**–**H**)) Mito-sox staining revealed that *myo*-inositol attenuated cisplatin-induced mitochondrial ROS generation in BUMPT cells (n = 4). (Panels (**I**–**L**)) C11 staining showed that *myo*-inositol alleviated cisplatin-induced lipid ROS generation in BUMPT cells (n = 4). (Panel (**M**)) MTT assay demonstrated that *myo*-inositol protected against RSL3-induced ferroptosis-related cell death in BUMPT cells (n = 6). (Panel (**N**)) RPKM value analyses showed that NOX4, among the seven known ferroptosis-related oxidases, has the highest expression in kidneys. (Panel (**O**)) Representative Western blots demonstrated that cisplatin treatment led to relative depletion of NCOA4 and FTH1 and up-regulation of NOX4, while *myo*-inositol treatment alleviated the changes in their expression. * *p* < 0.05 compared with control group, ^#^ *p* < 0.05 compared with CP group, scale bar: 50 μm.

To establish the relationship between the shielding effect of *myo*-inositol and ferroptosis, RSL3, a ferroptosis inducer with GPX4 inhibitory activity, was used in the present studies. MTT analysis revealed that *myo*-inositol treatment partially attenuated RSL3-induced ferroptosis in BUMPT cells (Figure 3M). Moreover, the status of oxidative stress, a key factor involved in ferroptosis, was evaluated by DCF and Mito-sox staining. We noted that cisplatin treatment led to a substantial increase in DCF- and Mito-sox-related fluorescence intensity in BUMPT cells (Figure 3B,F vs. Figure 3A,E and Figure 3D,H). Interestingly, the increased fluorescence was mitigated by the *myo*-inositol treatment (Figure 3C,G vs. Figure 3B,F and Figure 3D,H). Lipid ROS generation, as evaluated by C11 staining, was also increased in cisplatin-treated BUMPT cells, which was attenuated by *myo*-inositol (Figure 3I,L). In addition, FerroOrange staining showed that cisplatin-induced increased labile iron concentration was alleviated by *myo*-inositol treatment (Supplemental Figure S2). It has been previously reported that seven oxidative enzymes are involved in the pathogenesis of ferroptosis, among which NOX4 conceivably has the highest expression in kidneys, as indicated by RPKM (reads per kilobase of transcript/per million map reads) values (Figure 3N). Interestingly, cisplatin treatment led to a relatively increased expression of NOX4 in BUMPT cells, as assessed by immunoblotting. This increase was attenuated by *myo*-inositol treatment (Figure 3O, lane 4). Ferroptosis is usually associated with the occurrence of ferritinophagy, a process in which NCOA4 targeted ferritin into lysosomes for degradation. Therefore, NCOA4 and FTH1 can be simultaneously degraded in the ferroptosis process. Within expectation, a simultaneous degradation of NCOA4 and FTH1 was observed in cisplatin-treated BUMPT cells, which was partially restored by *myo*-inositol treatment (Figure 3O, lanes 1 and 2). All in all, these in vitro data indicated that *myo*-inositol down-regulates the expression profile of NOX4 to decelerate ferroptosis in cisplatin-treated BUMPT cells.

### 3.4. Myo-Inositol Promotes CHIP-Mediated NOX4 Ubiquitination to Decelerate Ferroptosis in Cisplatin-Treated BUMPT Cells

**Figure 4:**
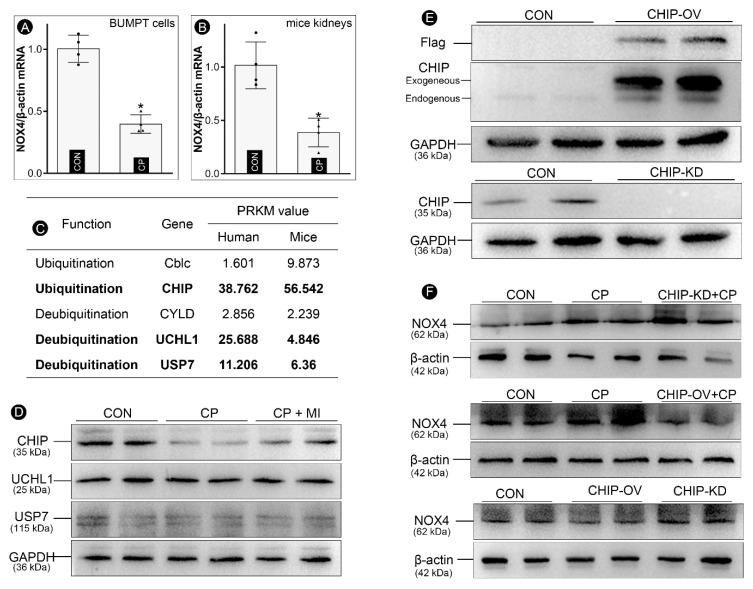
***Myo*-inositol promotes CHIP-mediated NOX4 in cisplatin-treated BUMPT cells.** (Panels (**A**,**B**)) qRT-PCR analysis showed that the mRNA levels of NOX4 have decreased in BUMPT cells and mice kidneys following cisplatin treatment (n = 4). (Panel (**C**)) RPKM value analysis demonstrated that CHIP, UCHL1, and USP7 have higher expression in the kidneys than the other two NOX4 ubiquitination-related enzymes. (Panel (**D**)) Representative Western blots revealed that CHIP was relatively depleted in cisplatin-treated BUMPT cells, and it was partially restored by *myo*-inositol treatment, while no obvious changes were observed in the expression of UCHL1 and USP7. (Panel (**E**)) Immuno-blotting analysis elucidating the overexpression and knockdown of CHIP in BUMPT cells. (Panel (**F**)) Immunoblot analysis revealed that the expression profile of CHIP modulated the protein levels of NOX4 in BUMPT cells. * *p* < 0.05 compared with control group.

To elucidate the mechanism involved in the up-regulation of NOX4 in the state of cisplatin-induced AKI, mRNA levels of NOX4 were evaluated in in vitro and in vivo studies. Interestingly, our qRT-PCR data revealed that NOX4 mRNA was decreased in cisplatin-treated BUMPT cells and mice kidneys (Figure 4A,B), indicating that post-translational events, not the transcriptional or post-transcriptional mechanism, are operationally responsible for the up-regulation of NOX4 (Figure 3O). Previous publications demonstrated that NOX4 can be degraded via a ubiquitin-dependent mechanism. There are five kinds of ubiquitination-related enzymes that have been described. The RPKM value analyses indicate that CHIP, UCHL1, and USP7 conceivably have relatively high expression in kidneys (Figure 4C). Furthermore, immune-blotting studies revealed that cisplatin treatment led to a down-regulation of CHIP, while no discernible changes in the expression of UCHL1 and USP7 were observed (Figure 4D). Interestingly, cisplatin-induced down-regulation of CHIP was attenuated by *myo*-inositol treatment in BUMPT cells (Figure 4D). In order to confirm the dominant role of CHIP in the expression profile of NOX4 in cisplatin-induced aberrations, CHIP-overexpression and CHIP-knockdown BUMPT cells were generated using lentivirus transfection, and the expression of CHIP was validated by immune-blotting analysis (Figure 4E). As expected, cisplatin-induced NOX4 up-regulation was accentuated by CHIP knockdown, but it was attenuated by its CHIP knockdown. Moreover, the expression of NOX4 could be modulated by CHIP in BUMPT cells without cisplatin challenge (Figure 4F). Taken together, our studies demonstrate that *myo*-inositol treatment up-regulates CHIP to attenuate the increased expression of NOX4, thus leading to the deceleration of ferroptosis.

### 3.5. Myo-Inositol Supplementation Attenuates Ferroptosis in Cisplatin-Induced AKI

**Figure 5:**
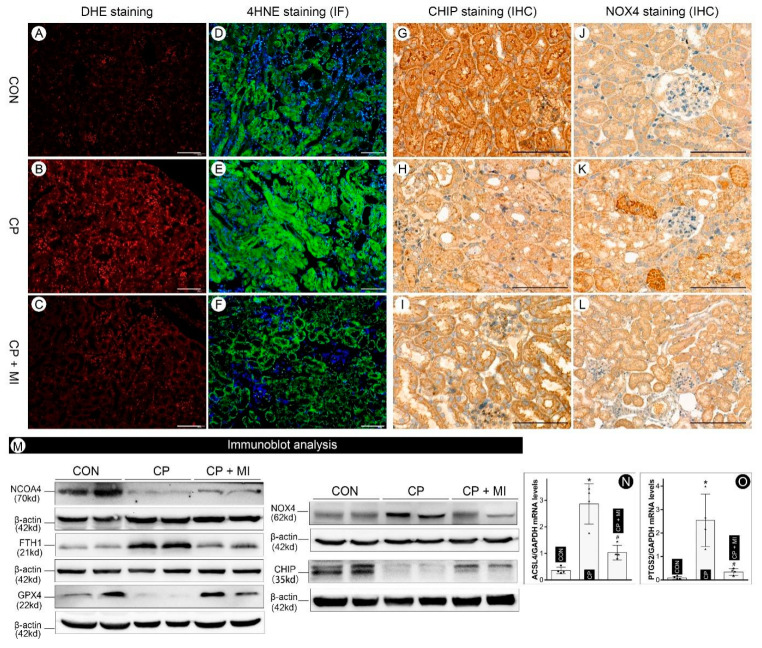
***Myo*-inositol alleviated ferroptosis in cisplatin-induced AKI in mice.** (Panels (**A**–**C**)) DHE staining showed that *myo*-inositol alleviates cisplatin-induced ROS generation in renal tubules. (Panels (**D**–**F**)) Immunofluorescence staining of 4-HNE revealed that the increased lipid peroxidation induced by cisplatin was attenuated by *myo*-inositol. (Panels (**G**–**L**)) Immunohistochemistry analyses demonstrated that CHIP was down-regulated while NOX4 was up-regulated in cisplatin-treated kidneys, and their expression was partially restored by *myo*-inositol treatment. (Panel (**M**)) Immunoblot analyses revealed that cisplatin treatment led to decreased expression of NCOA4, GPX4, and CHIP, while the expression of FTH1 and NOX4 increased. These changes were largely normalized with the supplementation of *myo*-inositol. (Panels (**N**,**O**)) qRT-PCR analysis showed that the mRNA levels of ACSL4 and PTGS2 have increased in mice kidneys following cisplatin treatment (n = 4), which was attenuated by *myo*-inositol treatment. * *p* < 0.05 compared with control group, ^#^ *p* < 0.05 compared with CP group, scale bar: 100 μm.

Multiple markers of ferroptosis were employed to delineate the modulation of ferroptosis by *myo*-inositol in cisplatin-induced AKI in mice. The reactive oxygen species (ROS) generation, as detected by DHE staining, was increased in cisplatin-treated kidneys, and their expression was attenuated by *myo*-inositol treatment (Figure 5A–C). Parallel immunofluorescence changes were observed in mice that had undergone various treatments following staining the kidney sections stained with 4-HNE, a marker of lipid peroxidation (Figure 5D–F). Moreover, our immuno-histochemistry studies also demonstrated that CHIP expression was down-regulated, while NOX4 was up-regulated in cisplatin-treated kidneys, and these changes were largely attenuated by *myo*-inositol supplementation (Figure 5G–L). Notably, the expression of both CHIP and NOX4 was mainly confined to the renal tubules (Figure 5G–L). The changes in CHIP and NOX4 expression were further validated by immune-blotting analysis (Figure 5M, right panel). In addition, other cisplatin-induced alterations, such as ferroptosis markers, including NCOA4, FTH1, and GPX4, were mitigated by *myo*-inositol treatment (Figure 5M, left panel). It is noteworthy that FTH1 was up-regulated in cisplatin-treated kidneys, unlike the changes in BUMPT cells. This unique change was induced by a feedback mechanism that the FTH1 mRNA was stabilized by increased labile iron in the ferroptosis states. Similar observations were reported in previous publications [14,26]. The expression of ACSL4 and PTGS2, two markers of ferroptosis, was also evaluated by qRT-PCR studies. Our work showed that cisplatin led to increased expression of ACSL4 and PTGS2, which was attenuated by *myo*-inositol treatment (Figure 5N,O). Overall, these data suggest that *myo*-inositol supplementation alleviates cisplatin-induced AKI via the inhibition of ferroptosis.

## 4. Discussion

The clinical use of cisplatin has been hampered by its dose-dependent side effects, e.g., nephrotoxicity, and the precise mechanism(s) related to such a cisplatin-induced acute kidney injury (AKI) remain elusive, despite many investigations that were devoted to this subject matter over a period of decades. Therefore, innovative exploratory efforts need to be made to unravel novel therapeutic agents that can dampen cisplatin-induced toxicity. Here, we present evidence that *myo*-inositol, an FDA-approved drug [27], may be an effective agent for reducing nephrotoxicity. The *myo*-inositol is a carbocyclic sugar that is involved in various biological processes, such as acting as a second messenger in diverse signaling events in mammalian systems [28]. It is noteworthy to point out that the biology of *myo*-inositol and the kidney is intricately intertwined since the kidney is one of the major sites for its de novo biosynthesis, especially in renal proximal tubules. It is estimated that about 4 mg of *myo*-inositol are synthesized by the two kidneys per day, which significantly exceeds the amount that is derived from a conventional diet (~1 mg/day) [29]. In addition, *myo*-inositol catabolism is exclusively confined to renal proximal tubular cells due to the specific expression of MIOX, a key enzyme that is responsible for the metabolic degradation of *myo*-inositol [23]. Our previous investigations revealed that genetic ablation of MIOX increases the concentration of serum and tissue *myo*-inositol in mice and attenuates cisplatin-induced AKI [24], suggesting that *myo*-inositol per se may participate in the modulation of renal injuries. In this study, we demonstrated that *myo*-inositol alleviated cisplatin-induced proximal tubular cell death in in vitro experiments, as indicated by the morphologic analyses and cell viability studies (Figure 1). In line with these observations, it was deduced that the aberrant alterations in renal injury-related physiological and biochemical parameters induced by cisplatin were conceivably attenuated by the supplementation of *myo*-inositol (Figure 2). Similarly, it has been reported that oral administration of *myo*-inositol attenuates redox injury and apoptosis in cadmium-induced nephropathy [25]. Furthermore, *myo*-inositol administration has been shown to modulate the osmoregulation in medullary tubules to alleviate C-methylene *myo*-inositol (MMI)-induced AKI [7]. Taking these literature data together, one may conclude that *myo*-inositol supplementation is effective in the attenuation of cisplatin-induced AKI.

Previously, our investigations demonstrated that ferroptosis is one of the critical bioprocesses in the progression of cisplatin-induced AKI, and the MIOX knockout, conceivably with high bodily concentration of *myo*-inositol, has been shown to exhibit deceleration of ferroptosis [14]. The pathogenesis of ferroptosis is closely interlinked with oxidative stress and perturbations in iron metabolism [30]. In this regard, our recently published data indicated that *myo*-inositol may serve as an important antioxidant involved in the modulation of redox injury in various pathobiological processes. Interestingly, the work of other investigators also reported that *myo*-inositol treatment alleviated oxidative stress in the sperm, ovary, heart, and liver [31,32,33]. Additionally, there are literature reports indicating that oral administration of *myo*-inositol partially restores the oxidative disruption of kidney homeostasis induced by cadmium [25]. However, the relationship between *myo*-inositol and iron metabolism has not been fully appreciated, although phosphate derivatives of *myo*-inositol have been implicated in various aspects of iron metabolism [34]. Given the above discussion, we addressed the question if *myo*-inositol can modulate the progression of ferroptosis in cisplatin-induced cellular injury. Our current studies revealed that *myo*-inositol treatment attenuated the cisplatin-induced aberrant ferroptosis-related cellular processes, including increased ROS generation (DCF and Mito-sox), lipid peroxidation (4-HNE), and ferritinophagy (NCOA4 and FTH1) (Figure 3 and Figure 5). Since various types of cell death have been ascribed to cisplatin-induced AKI, we also used RSL3, a unique ferroptosis inducer, to confirm if the ferroptosis “specifically” can be modulated by the *myo*-inositol treatment. As expected, the MTT assay demonstrated that ferroptosis-specific BUMPT cell death caused by RSL3 was, to a certain extent, mitigated by *myo*-inositol treatment (Figure 3). All in all, these observations suggested that *myo*-inositol decelerated ferroptosis to alleviate cisplatin-induced AKI.

The next pending issue is how *myo*-inositol regulates ferroptosis. As mentioned above, *myo*-inositol conceivably has biological properties to reduce oxidant stress and possibly lipid peroxidation; both being the core process of ferroptosis, we thus speculated that *myo*-inositol might be involved in the modulation of lipid peroxidation in cisplatin-induced AKI. In this scenario, lipid peroxide acts as the “executor” of ferroptosis, since its end products following decomposition are lethal to cells [35]. Previous literature indicates that lipid peroxide can be synthesized via iron-catalyzed Fenton reaction or lipid peroxidase-induced enzymatic reaction [30]. In this regard, seven different types of oxidases, including NOX1-4, ALOX5, ALOX12, and ALOX15, have been described that are operative during ferroptosis, among which NOX4 has the highest expression in the kidneys. Moreover, NOX4 is mainly expressed in the renal proximal tubular cells [36], where maximal renal damage is observed in cisplatin-induced AKI. Our present study demonstrated cisplatin treatment led to a substantial increase in NOX4 expression in the renal tubules (Figure 3). Similar results were reported by Meng et al., and the authors reported that NOX4 is up-regulated with accentuation of renal injury induced by cisplatin [37]. Interestingly, *myo*-inositol treatment partially offset the increased NOX4 expression induced by cisplatin, indicating that the inhibition of ferroptosis by *myo*-inositol conceivably is relevant to NOX4-catalyzed lipid peroxidation. 

Next, we further explored the mechanisms involved in the aberrant expression of NOX4. NOX4 is the major isoform of NADPH oxidase in kidneys, which produces H_2_O_2_ to participate in various physiological or pathological processes [38]. Previous publications have revealed that NOX4 overexpression exacerbated tubular damages in diabetic nephropathy [24], ischemia/reperfusion- or cisplatin-induced AKI [37,39], obstructive nephropathy [40], and hypertensive nephropathy [41]. Thus, NOX4 can be up-regulated in various scenarios, which might be dependent upon its transcriptional or post-translational events. Our present investigation noted that NOX4 mRNA was not upregulated in cisplatin-treated kidneys, suggesting that post-transcriptional mechanisms are most likely operative in the up-regulation of NOX4 protein. Some of the previous literature studies reported that ubiquitination-dependent degradation was intimately relevant to the protein expression levels of NOX4 [42]. Multiple enzymes, including two E3 ubiquitin ligases (CHIP and Cblc) and three deubiquitinases (CYLD, UCHL1, and USP7), were reported to modulate the stability of NOX4 [43,44,45,46]. RPKM value analyses revealed that CHIP, UCHL1, and USP7 have the highest expression in the kidneys (Figure 4). Interestingly, our immune-blotting analyses demonstrated that only CHIP conceivably has the most notable changes in its expression (up- or down-regulation) following treatment of cisplatin or cisplatin + *myo*-inositol. Notably, our additional immune-blotting studies pertaining to the up-or down-regulation of CHIP expression with concomitant remarkable up- or down-regulation of NOX4 suggest a dominant role of the latter in cisplatin-induced nephrotoxicity confined to the tubular compartment (Figure 4, panel F). Overall, these observations indicate that *myo*-inositol promotes CHIP-mediated NOX4 ubiquitination to decelerate the process of ferroptosis in cisplatin-induced AKI.

In summary, our present studies demonstrated that *myo*-inositol treatment inhibited ferroptosis to attenuate cisplatin-induced AKI, and this beneficial effect seems to be relevant to CHIP-mediated NOX4 ubiquitination and degradation. More importantly, these findings provide a novel therapeutic strategy to mitigate renal tubular injuries in various pathobiological processes.

## Supplementary Materials

The following supporting information can be downloaded at: https://www.mdpi.com/article/10.3390/cells12010016/s1, Figure S1: Identification of 10 mM *myo*-inositol for in vitro use; Figure S2: FerroOrange staining of BUMPT cell.
