# Supplementary material for: Myo-Inositol Supplementation Alleviates Cisplatin-Induced Acute Kidney Injury via Inhibition of Ferroptosis
Source: Cells. 2022 Dec 21;12(1):16. doi: 10.3390/cells12010016 (PMC9818458; doi:10.3390/cells12010016)
Supplement: Supplementary file 1 [file cells-12-00016-s001.zip › cells-2016852-supplementary.pdf]

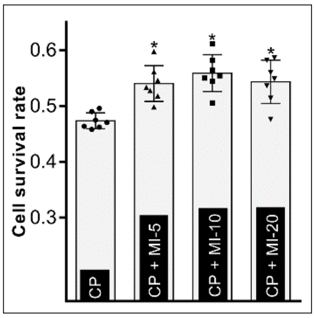

Supplemental figure S1. Identification of 10 mM myo-inositol for in vitro use.

MTT assay showed that 10 mM myo-inositol has better protection than that of 5 mM and 20 mM. MI-5: 5 mM myo-inositol, MI-10: 10 mM myo-inositol.*p<0.05.

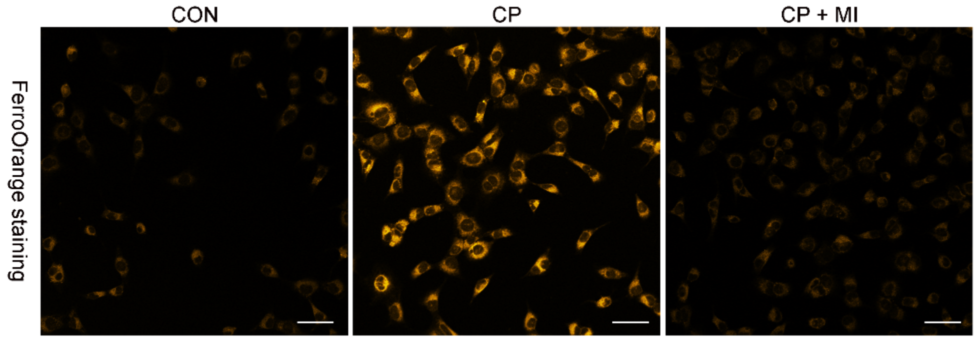

Supplemental figure S2. FerroOrange staining of BUMPT cells.

FerroOrange staining showed that cisplatin treatment lead to increased concentration of labile iron in BUMPT cells, which was attenuated by myo-inositol treatment.scale bar: 50 μm.
